# Cerebral responses to acupuncture for uremic pruritus: study protocol for a randomized controlled trial based on fNIRS

**DOI:** 10.3389/fnhum.2026.1892398

**Published:** 2026-08-28

**Authors:** Yuzhu Qu, Kongmei Ou, Runjia He, Yiting Wang, Xinyue Zhang, Yani Wang, Jie Chen, Benxiang He

**Affiliations:** 1 Post‑Doctoral Scientific Research Workstation of Affiliated Sport Hospital, Affiliated Sport Hospital of Chengdu Sport University, Chengdu, Sichuan, China; 2 Acupuncture and Tuina School, Chengdu University of Traditional Chinese Medicine, Chengdu, Sichuan, China; 3 Key Laboratory of Acupuncture for Senile Disease (Chengdu University of Traditional Chinese Medicine), Ministry of Education, Chengdu, Sichuan, China; 4 Hemodialysis Center, Chengdu Pidu District Hospital of Traditional Chinese Medicine, Chengdu, Sichuan, China; 5 Institute of Clinical Basis and Literature Information of Traditional Chinese Medicine, Sichuan Academy of Chinese Medicine Sciences, Chengdu, Sichuan, China

**Keywords:** acupuncture, antipruritus, central responses, functional near-infrared spectroscopy, uremic pruritus

## Abstract

**Introduction:**

Uremic pruritus (UP) is a common complication of end-stage renal disease that significantly impairs patients’ quality of life. Although acupuncture can effectively relieve UP symptoms, its central mechanism remains unclear.

**Methods and analysis:**

A total of 96 patients with UP will be randomly assigned to three groups: verum acupuncture (VA), sham acupuncture (SA), or waiting-list (WT) control. All participants will undergo a cowhage test to induce acute itch and receive a single session of instant VA, SA, or no intervention (WT) during functional near-infrared spectroscopy (fNIRS) scanning. Subsequently, participants in the VA and SA groups will receive 4 weeks of consecutive acupuncture treatment. Hemodynamic changes in the prefrontal cortex and primary somatosensory cortex will be monitored during the instant acupuncture session, as well as after the 6^th^ and 12^th^ sessions of long-term acupuncture. Clinical outcomes involving itch intensity, quality of life, and psychological status will also be assessed.

**Discussion:**

This study aims to explore the central mechanisms underlying both the immediate and long-term effects of acupuncture on UP in a real clinical setting using fNIRS. We hypothesize that VA will specifically modulate neural activity patterns in itch-related brain regions, and that these changes will correlate with clinical symptom improvement. This study will provide visual neuroimaging evidence for the antipruritic effects of acupuncture, thereby facilitating more precise acupuncture treatments for UP.

**Clinical trial registration:**

https://itmctr.ccebtcm.org.cn, identifier ITMCTR2025001844.

## 1 Introduction

Uremic pruritus (UP) is a prevalent and distressing complication of end-stage renal disease (ESRD), affecting approximately 40–90% of patients undergoing maintenance hemodialysis (Sanchez-Alvarez et al., 2024). UP is characterized by persistent or paroxysmal severe itching, that profoundly impairs sleep quality, emotional well-being, and social functioning, thereby reducing patients’ quality of life; it is also associated with increased mortality risk (Tennankore et al., 2024). Current therapeutic options for UP include antihistamines, topical emollients, phototherapy, gabapentin or pregabalin, and opioid receptor modulators (e.g., difelikefalin, nalfurafine). These treatments have limitations in efficacy, adverse effects, or accessibility (Kljajić et al., 2026), underscoring the need for safe and effective complementary or alternative therapies.

Acupuncture, a core component of traditional Chinese medicine, has been shown to effectively alleviate UP symptoms in numerous clinical studies, including reducing pruritus severity and improving sleep and mood (Yeam et al., 2021; Zhang et al., 2023). Additionally, some research suggests that its benefits for UP in patients with ESRD are related to the regulation of peripheral immune responses, including decreasing serum levels of interleukin-2, histamine, and 5-hydroxytryptamine (Ardinata et al., 2022; Gan et al., 2023). Although our previous studies have highlighted the importance of central integration in the effects of acupuncture (Yin et al., 2023a; Yin et al., 2023b), the central mechanisms underlying its therapeutic effects on UP remain unclear. Understanding how acupuncture modulates brain activity during clinical treatment is important for optimizing therapeutic strategies. Therefore, it is necessary to explore the central mechanisms of its antipruritic effects on UP.

Functional near-infrared spectroscopy (fNIRS) offers advantages for investigating acupuncture’s neural mechanisms in real clinical settings. This non-invasive technique tracks cortical hemodynamic responses by detecting oxygenated and deoxygenated hemoglobin concentrations based on the neurovascular coupling principle. Compared to functional magnetic resonance imaging (fMRI), fNIRS is portable, resistant to motion artifacts, and cost-effective. It also allows patients to receive acupuncture in a natural sitting posture, making it particularly suitable for real-time monitoring in real-world clinical settings (Xu et al., 2025). Compared with electroencephalography (EEG), fNIRS provides better signal stability and spatial accuracy (Wang et al., 2020). Furthermore, fNIRS has been successfully employed to investigate individual central responses to acupuncture in natural clinical environments (Cao et al., 2026; Qu et al., 2026).

Accordingly, this randomized controlled trial aims to elucidate the central mechanism of acupuncture for UP by using fNIRS to assess brain responses to immediate and long-term acupuncture interventions. We hypothesize that, compared to sham acupuncture (SA) and waiting-list (WT) controls, verum acupuncture (VA) will specifically regulate neural activity patterns in the prefrontal cortex (PFC) and primary somatosensory cortex (S1) in response to itch. We further hypothesize that these neural changes will correlate with clinical symptom improvement. Therefore, this study aims to elucidate the central mechanism of acupuncture for UP, thereby providing insights into the scientific basis of acupuncture for UP and supporting its precise clinical application.

## 2 Methods and analysis

### 2.1 Study design

**Figure 1:**
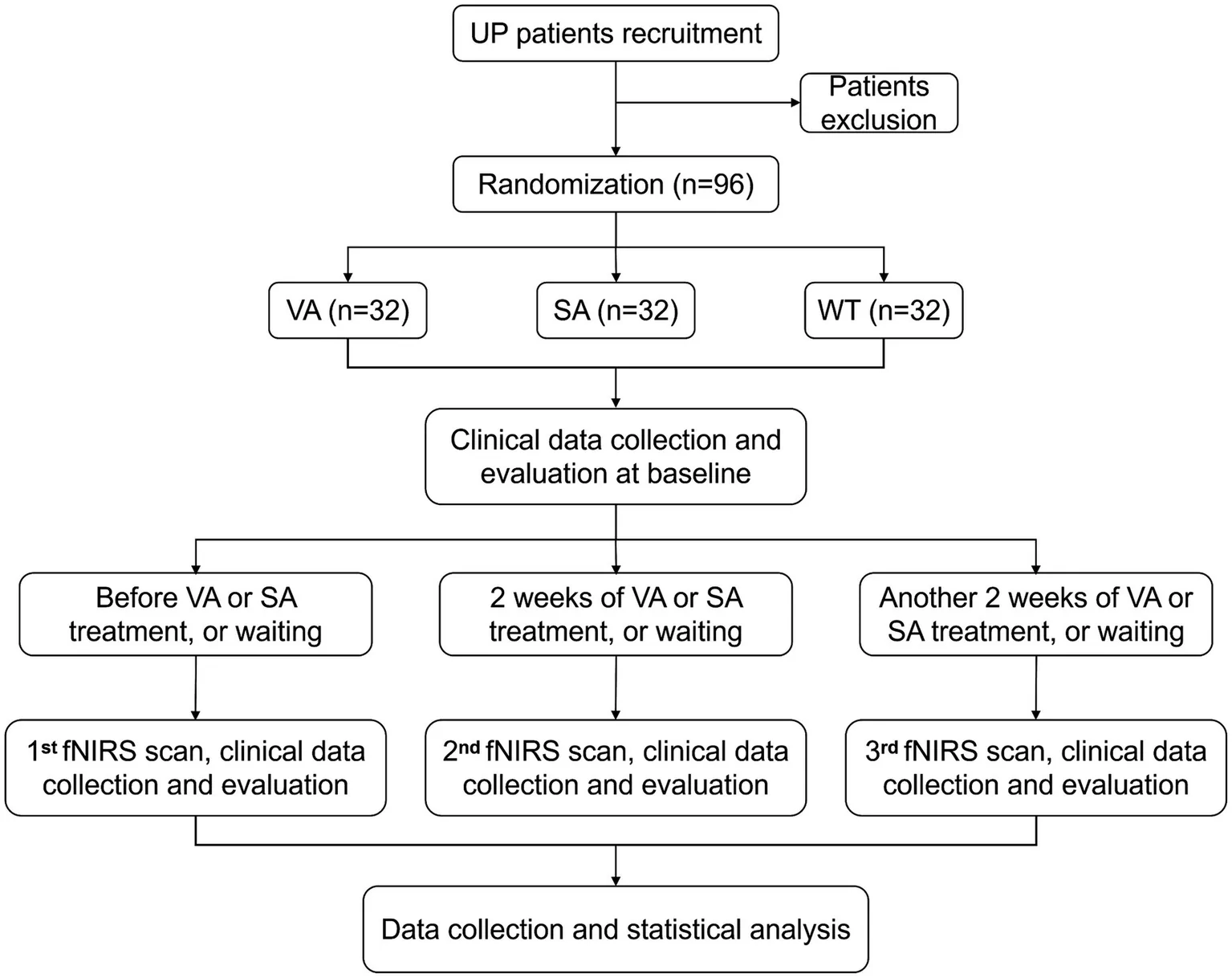
Flow chart of the trial. VA, verum acupuncture; SA, sham acupuncture; WT, waiting-list; fNIRS, functional near-infrared spectroscopy.

This is a randomized clinical trial based on fNIRS, and a total of 96 patients with UP will be enrolled. Patients will be randomly divided into three groups (VA, SA, or WT groups) in a ratio of 1:1:1 after meeting the inclusion criteria. Participants in the VA and SA groups will receive one session of immediate verum or sham acupuncture during scanning, followed by 4 weeks of long-term verum or sham acupuncture treatment, respectively. The clinical evaluation and fNIRS scans are presented in Figure 1 and Table 1. The study is following the Standard Protocol Project Items: Recommendations for Intervention Trials (SPIRIT) guidelines, the principles of Consolidated Standards of Reporting Trials (CONSORT), and the Standards for Reporting Interventions in Clinical Trials of Acupuncture (STRICTA).

**Table 1:** Schedule of enrollment, interventions, fNIRS scan, and assessment.

Time point	Study period					
	Baseline		Treatment phase			
	1-week	0-week	1-week	2-week	3-week	4-week
Enrollment						
Eligibility screen	×					
Informed consent	×					
Randomization		×				
Interventions						
VA group			×	×	×	×
SA group			×	×	×	×
WT group			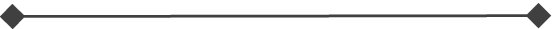			
fNIRS Scan						
VA group		×^*^		×		×
SA group		×^*^		×		×
WT group		×^*^		×		×
Assessment						
VAS of cowhage induced itch			×			
24 h-WI-NRS		×	×	×	×	×
UP-dial		×				×
PHQ-9		×				×
GAD-7		×				×
Adverse events		×	×	×	×	×
Blinding						×^**#**^

*means the first fNIRS scan will be performed for both measuring the immediate antipruritic effect of acupuncture induced by the cowhage test and the long-term effects before the 4-weeks treatment. #means after completing the 4-week trial and final clinical evaluation, participants in the VA and SA groups will be asked to guess whether they received verum acupuncture, sham acupuncture, or were unaware of it. VA, verum acupuncture; SA, sham acupuncture; WT, waiting-list; fNIRS, functional near-infrared spectroscopy; VAS, visual analogue scale; 24 h-WI-NRS, 24-h Worst Itch Numeric Rating Scale; UP-Dial, Uremic Pruritus in Dialysis patients; PHQ-9, Patient Health Questionnaire-9; GAD-7, Generalized Anxiety Disorder Scale-7.

### 2.2 Ethics

This study has been approved by the Institutional Review Board and Ethics Committee of Chengdu Pidu District Traditional Chinese Medicine Hospital (No. K-2025-011) and registered on September 26, 2025, in the International Traditional Medicine Clinical Trial Registry of World Health Organization, an international clinical trial registration platform (ITMCTR2025001844).

### 2.3 Recruitment

This study will be carried out from March 2026 to December 2027 at Chengdu Pidu District Traditional Chinese Medicine Hospital. UP patients will be recruited from the blood purification center of this hospital.

### 2.4 Participants

#### 2.4.1 Diagnostic criteria

UP is diagnosed according to the ESRD criteria, characterized by itching that cannot be attributed to other causes (Fishbane et al., 2020a, 2020b). Based on the KDIGO 2024 Clinical Practice Guidelines for the Evaluation and Management of chronic kidney disease, patients with eGFR<15[ml·min^−1^(1.73 m^2^)^−1^ sustained for ≥ 3 months meet the diagnostic criteria for ESRD (Kidney Disease: Improving Global Outcomes (KDIGO) CKD Work Group, 2024).

#### 2.4.2 Inclusion criteria

Patients who match all the following items will be included:1) Meet the diagnostic criteria for UP and lasts for 6 weeks or longer, and 2) moderate to severe itch of UP (average weekly score of 24-h Worst Itch Numeric Rating Scale (24 h WI-NRS) in the past week≥ 4 points), and 3) aged 18 to 65 years with right-handed, and 4) have been regularly undergoing hemodialysis for more than 3 months, and 5) within the first 3 months before enrollment, at least one single chamber urea clearance rate (spKt/V) of ≥ 1.2 and urea decline rate (URR) of ≥ 65%, and 6) not combined with serious mental illness or cognitive impairment, and 7) not combined with neurological diseases such as cerebral hemorrhage and cerebral infarction, and 8) without alcohol or drug addiction, 9) medications for UP, such as antihistamines, gabapentin, topical emollients, and other antipruritic agents, need to maintain a stable dosage for at least 1 month before enrollment, and any newly increased antipruritic drugs are forbidden during the trial, and 10) not participating in other clinical trials currently, and 11) sign informed consent before conducting the trial.

#### 2.4.3 Exclusion criteria

Patients will be excluded if they:1) have other skin diseases that can cause itching, such as psoriasis, atopic dermatitis, urticaria, contact dermatitis, etc., and 2) suffer from any severe chronic liver disease, or any one of alanine aminotransferase, aspartate aminotransferase, glutamyltransferase, or total bilirubin exceeding twice the upper limit of normal values, and 3) with malignant tumors or other serious illnesses that require hospitalization, and 4) with severe mental illness or cognitive impairment (such as dementia), and 5) only experience itching during hemodialysis, and 6) have undergone physical therapy such as acupuncture or moxibustion within 1 month to address itching symptoms, and 7) present with coagulation dysfunction or local skin infection and ulceration, and 8) pregnant or lactating women.

### 2.5 Sample size

Since this trial will take the therapeutic effects of acupuncture on UP as the foundation and further explore its central mechanism via fNIRS, 24 h-WI-NRS will be used as the primary clinical outcome for this sample size calculation (Fishbane et al., 2020a, 2020b), with VA versus SA defined as the primary comparison; meanwhile, the WT group will serve as an auxiliary control to separate spontaneous disease fluctuation. Based on our preliminary pilot trial, the 24 h-WI-NRS mean differences were 3.68 ± 1.30 for the VA group and 1.64 ± 2.55 for the SA group after 4-week interventions compared to baseline. Using G*Power software with two-tailed tests, effect size = 1.01, *α* = 0.05, and 1-*β* = 0.95, this study requires 27 patients per group. Accounting for a 15% dropout rate, each group should enroll 32 patients; a total of 96 patients will be included across three groups.

### 2.6 Randomization

To avoid selection bias, the randomization will be performed with a computer-generated randomization digital table to assign these 96 patients into VA, SA, and WT groups in a 1:1:1 ratio. The sealed envelopes will be used to inform the acupuncturist what kind of acupuncture the patient will receive.

### 2.7 Blinding

Since the acupuncturist is always aware of the grouping of patients, the acupuncture manipulation will be concealed from participants, researchers, outcome evaluators, and statisticians. Researchers, the acupuncturist, outcome evaluators, and statisticians are separate. To minimize non-specific effects arising from acupuncturist-participant interaction, a standardized communication script will be implemented across all groups, restricting conversation to neutral, pre-specified phrases (e.g., greeting, procedural instructions, and safety checks only). To assess blinding success, after completion of the 4-week trial and final clinical assessment, participants in the VA and SA groups will be asked to guess whether they received verum acupuncture, sham acupuncture, or were unaware of it; the Bang blinding index will then be calculated for these two groups.

### 2.8 Cowhage test for inducing pruritus

The primary neuronal transmission pathway of UP is mediated by non-histamine pathways. Hence, the cowhage test could induce acute pruritus in UP patients (Papoiu et al., 2014). According to previous studies (Zuo et al., 2023), about 20 cowhage spicules will be applied using a gloved finger to a 2 cm diameter area located about 5 cm below the elbow of the patient’s non-fistula forearm. The spicules will then be gently rubbed on the skin in a circular motion for 30 s. The itch-inducing procedure will take approximately one minute. Pruritus intensity will be assessed every minute for 5 min using a 0–10 visual analogue scale (VAS). After the test, spicules will be completely removed using adhesive tape.

### 2.9 Acupuncture interventions

After enrollment, patients in the VA and SA groups will receive an immediate acupuncture treatment for cowhage-induced pruritus during fNIRS scanning, followed by a long-term treatment of 12 sessions over 4 weeks (three sessions per week). Acupuncture points (Kim et al., 2010) in this trial include the bilateral: *Quchi* (LI11), *Hegu* (LI4), *Xuehai* (SP10), *Sanyinjiao* (SP6), *Zusanli* (ST36), and *Shenshu* (BL23).

#### 2.9.1 Instant acupuncture treatment

The instant acupuncture will be performed at acupoints using disposable sterile filiform needles (0.25 × 40 mm, Huatuo Medical Instrument Co., Ltd., China). In the VA group, the acupuncturist will remove the rubber ring from the patient’s skin at the base of the Park Sham Placebo Acquire Device (PSD) and attach the plastic sleeve to the acupoint surface. The needles will then be retained in the acupoints for 5 min during fNIRS scanning. In the SA group, patients will receive acupuncture using the PSD with blunt needles. Once the needle touches the skin, it will retract into the plastic sleeve of the PSD. No additional acupuncture manipulations will be performed, and the needle will remain on the skin for 5 min during fNIRS scanning.

#### 2.9.2 Long-term acupuncture treatment

##### 2.9.2.1 VA group

The PSD will also be used during long-term acupuncture treatment. After skin disinfection using alcohol, needles will be inserted to a depth of 20-30 mm. The acupuncturist will then twist the needle (90°-180°) and perform lift-thrust manipulations with an amplitude of 3-5 mm at a frequency of 1–1.5 Hz to induce needle sensations (soreness, numbness, distention, and heaviness). Each 30 min session includes 10–15 s of needle manipulation every 10 min to sustain the needle sensation.

##### 2.9.2.2 SA group

Patients in this group will receive treatment at identical acupoints as the VA group. While these patients’ skin will not be actually punctured by using blunt needles with PSD. During 30 min treatment, any other acupuncture manipulation will not be performed.

##### 2.9.2.3 WT group

Patients assigned to the WT group will not receive any acupuncture treatment at the cowhage test or during the 4-week trial. After completing the trial, they can voluntarily choose whether to accept 12 sessions of free acupuncture treatment provided by our research group. To alleviate the impact of attention/expectation effects from clinical contact, WT participants will receive weekly telephone symptom monitoring by a blinded researcher. Retention strategies for this group include flexible scheduling for the post-trial free acupuncture, regular follow-up telephone calls, and transportation reimbursement.

### 2.10 Clinical assessments

#### 2.10.1 Instant itch intensity induced by the cowhage test

Patients will use VAS to rate itch intensity induced by the cowhage test during the first fNIRS scanning, with an evaluation per minute. The VAS scale ranges from 0 ~ 10, and the end-points anchoring the VAS are: 0 = no itch sensation, and 10 = maximum unbearable itch. The peak VAS itch score recorded during the observation period will be extracted for statistical analysis.

#### 2.10.2 Primary clinical outcome

The weekly mean 24 h-WI-NRS scores at baseline and during each week of the 4-week trial period will be evaluated as the primary clinical outcome (Fishbane et al., 2020a, 2020b). The 24 h-WI-NRS scale ranges from 0 (no itch sensation) to 10 (maximum unbearable itch) points.

#### 2.10.3 Secondary clinical outcomes

##### 2.10.3.1 14-item uremic pruritus in dialysis patients scale

Patients will use the 14-Item Uremic Pruritus in Dialysis Patients Scale to evaluate the quality of life from a multi-dimensions such as symptoms of pruritus, social psychology, and sleep, comprehensively reflecting their quality of life (Nochaiwong et al., 2024). The scores ≤12 mean mild damage, scores 13 ~ 21 represents moderate damage, and scores ≥22 represent severe damage to the quality of life. The scores at week 4 and the baseline period will be used for statistical analysis.

##### 2.10.3.2 Psychological condition

Since patients with UP are frequently accompanied by mental disorders (Faucon et al., 2025), the Patient Health Questionnaire-9 (PHQ-9) and Generalized Anxiety Disorder Scale-7 (GAD-7) will be used to evaluate their emotional state before and after this trial. PHQ-9 score ranges from 0 ~ 4, 5 ~ 9, 10 ~ 14, 15 ~ 19, and 20 ~ 27, corresponding to no, mild, moderate, moderately severe, and severe depression, respectively. GAD-7 score ranges from 0 ~ 4, 5 ~ 9, 10 ~ 14, and≥15, being classified as no, mild, moderate, and severe anxiety, respectively.

### 2.11 Core neuroimaging indicator: fNIRS data acquisition

Based on the efficacy of acupuncture for UP, this study will further focus on its central mechanism. According to previous studies (Mochizuki and Kakigi, 2015; Sun, 2025), we hypothesize that acupuncture’s antipruritic effect of UP correlates with modulation of cortical regions involved in itch processing, particularly the prefrontal cortex (PFC) and primary somatosensory cortex (S1), which can be captured in real-time by portable fNIRS. Moreover, considering that the oxyhemoglobin (HbO) signal has a better signal-to-noise ratio and is more reliable and sensitive than the deoxyhemoglobin signal (Strangman et al., 2002; Dravida et al., 2018), this trial will use cortical HbO changes to characterize the functional activity of PFC and S1, two regions of interest (ROIs). Therefore, the HbO change of task-state scanning data during the first fNIRS scan will be the primary outcome of the immediate effect of acupuncture on relieving itch, and the resting-state scanning data after 0−/2−/4-week interventions will be the primary mechanistic outcome of acupuncture’s long-term effect on UP.

**Figure 2:**
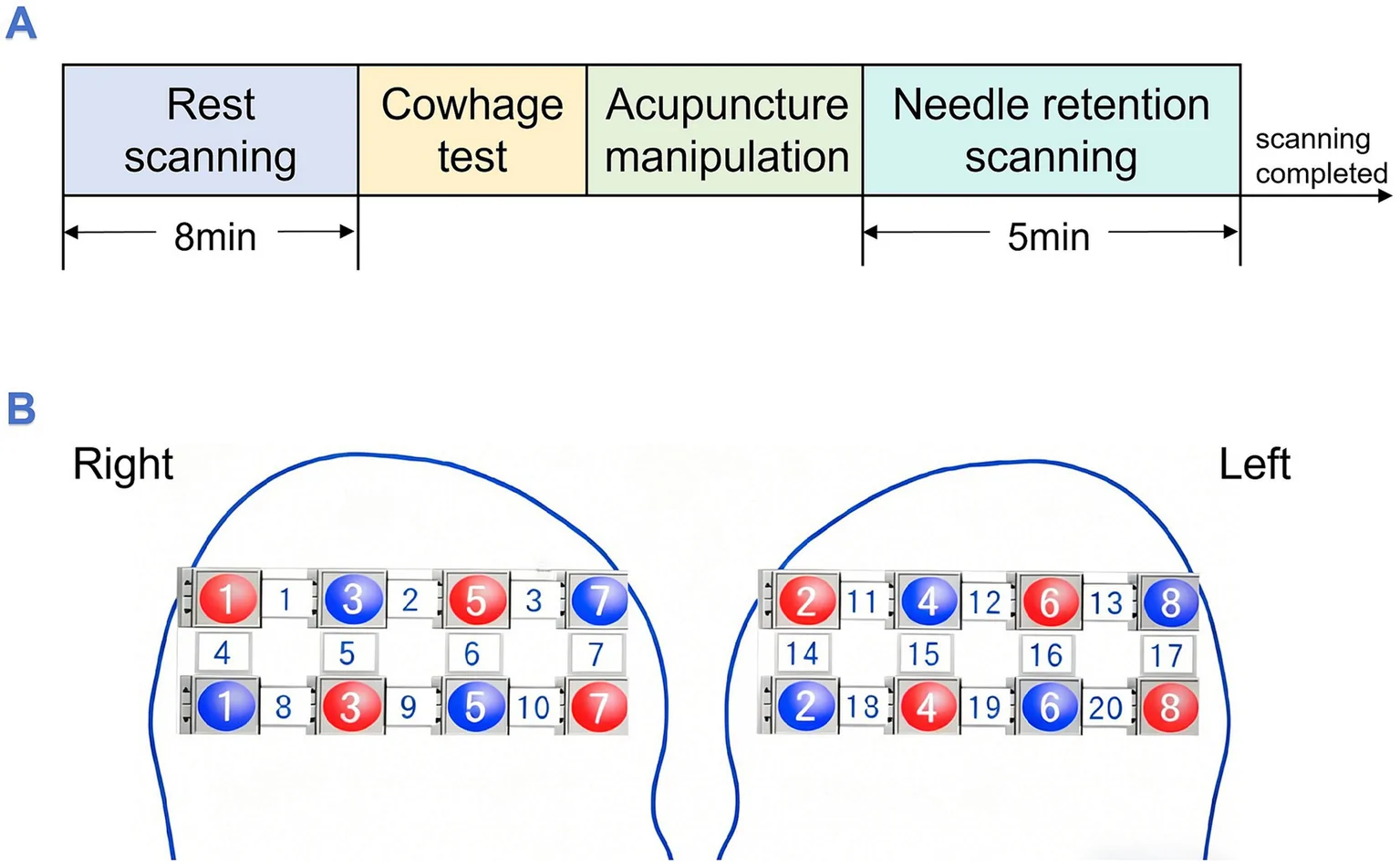
fNIRS data acquisition. **(A)** fNIRS scanning process diagram; **(B)** fNIRS channels location.

Patients will undergo three fNIRS recording sessions. Each fNIRS scan will be scheduled on non-dialysis days or before dialysis sessions to minimize the influence of acute biochemical fluctuations. The first fNIRS scan will be conducted before the long-term acupuncture treatment, including an 8 min rest scanning and a 5 min task scanning during acupuncture treatment with immediate itch induction by the cowhage test (Figure 2A). Another two fNIRS scans will be performed after the 6th and 12th long-term acupuncture treatments, with only the 8 min rest scanning.

A portable fNIRS instrument (LIGHTNIRS, Shimadzu Corporation, Japan) will be utilized to measure cortical hemodynamic response, with a sampling rate of 13.33 Hz at wavelengths 780 nm and 830 nm. Due to PFC and S1 being ROIs of the mechanism hypothesis in this study, the optodes of fNIRS will be positioned over the prefrontal and bilateral primary somatosensory cortex. The fNIRS cap is created by 8 × 2 light sources and detectors (20 channels) placed according to the international 10–20 EEG system, with a separation distance of 2.5–3 cm for all optodes (Figure 2B). To achieve precise spatial registration, the Montreal Neurological Institute coordinates of all optodes will be measured using an electromagnetic 3D digitizer system, and the corresponding brain regions of the midpoint of each channel in the Brodmann Talairach template will be calculated.

### 2.12 Data management

Clinical data will be managed through both paper and electronic case report forms (CRFs). Only outcome evaluators have access to CRFs and will perform double-data entry. The Evidence-based Medicine Center of the Affiliated Third Hospital of Chengdu University of Traditional Chinese Medicine will supervise the study and monitor the data regularly.

### 2.13 Patient safety

In order to evaluate the risks caused by acupuncture, the safety assessment will be conducted after each acupuncture session. Adverse events such as hematoma, bleeding, severe pain, infection, fainting, or other severe events will be processed immediately, recorded with details in CRFs, and monitored by the Ethics Committees.

### 2.14 Data analysis

#### 2.14.1 Clinical data analysis

The primary clinical analysis will strictly follow the intention-to-treat (ITT) principle. Missing data will be handled using multiple imputation (MI) under the missing-at-random assumption, and sensitivity analyses will include per-protocol (PP) and complete-case analyses. The clinical data will be analyzed using SPSS 22.0 software. For the primary outcome (weekly mean 24 h-WI-NRS scores), a linear mixed-effects model (LMM) will serve as the primary analytic approach, including time, group, and their interaction as fixed effects, and a random intercept for each participant. In this primary model, the baseline measurement will be treated as the initial level of the time factor (Time = 0) rather than as a covariate, to avoid model misspecification and biased estimates. A pre-specified sensitivity analysis will additionally adjust for baseline outcome values and key demographics (age and sex) to test the robustness of the primary findings.

For secondary outcomes (measured at baseline and week 4), LMM will also be applied, with the same fixed and random effects structure as described above. If the LMM residuals violate normality assumptions, non-parametric alternatives (Wilcoxon signed-rank test for within-group comparisons; Kruskal-Wallis and Dunn test for between-group comparisons) will be applied. For the single-timepoint continuous data (instant cowhage-induced itch VAS, assessed only during the first fNIRS scanning), one-way ANOVA will be used if the data follow a normal distribution, or the Kruskal-Wallis test if not. To correlate fNIRS results with clinical outcomes, Spearman rank correlation will be used as a non-parametric alternative to Pearson correlation if normality assumptions are violated, and the false discovery rate (FDR) correction will be applied for multiple comparisons. The counting data will be analyzed using the chi-square test. The statistical significance threshold is set at 0.05 with the two-sided test.

#### 2.14.2 fNIRS data analysis

The fNIRS data will be processed and analyzed using the MATLAB 2020b software.

In lines with prior fNIRS studies investigating acupuncture (Qu et al., 2022; Si et al., 2021), fNIRS data preprocessing will be implemented using the Homer2 toolbox, which mainly including: (1) noisy channels will be rejected if any of the following criteria are met: the optical intensity (OI) falls outside the range of 0.5–1,000, the source-detector separation deviates over the range from the 0–45 mm (Broscheid et al., 2022), or the ratio between the mean optical intensity and its standard deviation [mean(OI)/std.(OI)] is less than 2; (2) at the participant level, if over 50% of a participant’s total channels are flagged as noisy, the entire dataset of this participant will be discarded due to insufficient signal quality; (3) the raw NIRS light intensity will be converted to an optical density (OD) signal; (4) the function hmrMotionArtifactByChannel will be used to identify motion artifacts; (5) then the motion correction will be conducted with the spline interpolation algorithm implemented in the function hmrMotioncorrectionSpline; (6) the interference of physiological noises will be reduced using a 0.01–0.2 Hz bandpass filter; and (7) the filtered OD data will be converted into the concentrations of HbO based on the modified Beer–Lambert law. Then, the hemodynamic response function will be estimated by a general linear model approach that uses ordinary least squares to get an average response for each of 20 CHs in every participant. Brain activation analysis will be conducted, and based on Montreal Neurological Institute (MNI) coordinates, CHs covering PFC and S1 will be predefined as the ROIs for subsequent functional connectivity analysis.

Given that the first fNIRS session includes both resting-state and task-state (cowhage-induced itch with VA or SA intervention, or WT control) scanning, while the 2−/4-Week-4 sessions only include resting-state scanning, these data will be analyzed separately according to different physiological states. Specifically, for the immediate effect of acupuncture antipruritus, the task-induced hemodynamic change (ΔHbO = task-state minus resting-state) during the first fNIRS scan will be calculated for each group. Since this is a single time-point measurement, the comparison (VA vs. SA vs. WT) of theseΔHbO values will be performed using one-way ANOVA. For the long-term effect of acupuncture antipruritus, only resting-state fNIRS data will be acquired at three time points (0−/2−/4-week). To account for the repeated-measures design across the three time points, LMM will be applied to longitudinal hemodynamic responses, with time, group, and their interaction as fixed effects, and a random intercept for each participant. The resting-state outcome measures will comprise: (1) regional HbO activity, defined as the temporal mean of the HbO concentration over the resting-state period for each channel; and (2) HbO-based functional connectivity, calculated as Fisher’s Z-transformed Pearson correlation coefficients between the time series of all channel pairs within and across the PFC and S1. The significance threshold is set to *p* < 0.05, and FDR correction will be strictly applied to control for multiple comparisons.

## 3 Discussion

This is the first fNIRS study to investigate cerebral responses to acupuncture for UP. It will explore the central mechanisms underlying acupuncture’s antipruritic effects. Hence, our findings may help elucidate the scientific basis of acupuncture for UP.

### 3.1 Reasons for grouping design and sham acupuncture selection

In this study, the SA and WT groups are designed to distinguish the specific therapeutic effects of VA from placebo responses and the natural fluctuation of UP. The PSD with blunt needles serves as a suitable sham acupuncture control, helping to maintain participant blinding and avoiding skin penetration (Lim and Go, 2024). Evidence indicates that non-penetrating sham acupuncture produces the smallest placebo effect, thereby optimizing the detection of non-specific effects (Wan et al., 2025). WT group is a design established for stable chronic conditions in randomized controlled trials of acupuncture (Hershman et al., 2018). This design accounts for spontaneous symptom variation, regression to the mean, and the natural course of UP without acupuncture intervention, while ensuring ethical compliance through the guaranteed offer of 12 sessions of free post-trial acupuncture. Additionally, weekly telephone symptom monitoring will be provided during the waiting period to balance clinical contact across the three groups. Since blinding of acupuncturists is not feasible, strict separation of roles among researchers, outcome assessors, and statisticians is essential to minimize bias in this trial.

### 3.2 Reasons for cowhage test selection

The cowhage test offers a consistent and reproducible method for inducing non-histaminergic pruritus, which better reflects the pathophysiology of chronic itch than histamine-induced itch (Mochizuki et al., 2015; Zuo et al., 2021). Neuroimaging studies have shown abnormal brain responses to cowhage-induced itch in UP patients, involving regions such as the PFC and S1 (Papoiu et al., 2014). Cowhage allows precise temporal alignment between itch induction, acupuncture intervention, and fNIRS recording, facilitating the analysis of neural responses at different stages. The inclusion of both instant and long-term (0−/2−/4-week) assessments enables differentiation between transient neuromodulatory effects and sustained neuroplastic changes.

### 3.3 Reasons for selecting fNIRS for detecting the cerebral responses to acupuncture

Choosing fNIRS as the neuroimaging modality represents a balance between scientific rigor and clinical practicality. Unlike fMRI, fNIRS is portable, quiet, and allows patients to receive acupuncture in a naturalistic position, with high tolerance for minor movements common among UP patients (Fu et al., 2023). Compared with EEG, fNIRS provides superior signal stability and more precise spatial localization (Li et al., 2022). Specifically, EEG relies on scalp electrodes to record electrical activity and is susceptible to interference from electrode impedance and sweat. Additionally, its source localization requires head model estimation, which limits accuracy (Azizollahi et al., 2023; Kalevo et al., 2020). In contrast, fNIRS measures blood oxygenation changes through optical signals and is minimally affected by scalp conditions; it also assesses cortical activity changes based on hemodynamic signals (Pereira et al., 2023). Meanwhile, a 16–20 channel montage is sufficient to cover the PFC and S1, which are key regions involved in itch processing and acupuncture modulation. These features enable real-time monitoring of cortical responses to acupuncture across different treatment stages, such as brain dynamics during cowhage-induced itch, during acupuncture or needle retention, and during long-term treatment at 0−/2−/4-weeks. Consequently, our analytical strategy will separate the task-evoked acute responses from the resting-state longitudinal changes, allowing us to explore the immediate neural regulatory effects and the sustained central plasticity induced by acupuncture treatment.

### 3.4 Limitations

However, several limitations should be acknowledged and managed. Firstly, uremic toxin level fluctuations in hemodialysis patients may affect cerebral hemodynamics. To address this, we will define strict dialysis adequacy criteria (spKt/V ≥ 1.2) in the inclusion criteria. Secondly, although the cowhage-induced itch model reliably activates non-histaminergic pruriceptive pathways relevant to UP, its intensity and temporal profile may differ from spontaneous uremic itch. Therefore, to address this limitation, we will perform additional analyses of resting-state brain activity and spontaneous itch perception during needle retention. In addition, with a detection depth of 20 mm approximately, fNIRS primarily measures cortical hemodynamics and cannot directly assess subcortical itch-related regions, such as the thalamus, insula, and brainstem. Future studies may utilize neuroimaging techniques with higher spatial resolution (e.g., fMRI) or multimodal approaches (e.g., fMRI-EEG) to further explore the central mechanisms of acupuncture for UP.

## 4 Conclusion

In summary, this study aims to elucidate the central mechanism of acupuncture for UP. The findings are expected to provide visual neuroimaging evidence for its antipruritic effects and may inform optimized combination strategies with existing pharmacotherapies, guiding more effective individualized treatment for UP.
